# Methods to model epicardial adipose tissue-mediated atrial fibrillation

**DOI:** 10.1038/s44325-025-00065-7

**Published:** 2025-06-26

**Authors:** LC Cook, MD Perry, AP Hill, J Thorpe

**Affiliations:** 1 Victor Chang Cardiac Research Institute, Darlinghurst, Australia; 2 School of Clinical Medicine, Faculty of Medicine and Health, UNSW, Sydney, Australia; 3 School of Biomedical Sciences, Faculty of Medicine and Health, UNSW, Sydney, Australia

**Keywords:** Stem cells, Atrial fibrillation, Cardiovascular diseases

## Abstract

Obesity is a major risk factor for atrial fibrillation (AF), partly mediated through an increased volume of epicardial adipose tissue (EAT) which directly interacts with the adjacent myocardium. However, the specific underlying mechanisms remain unclear. This narrative review explores animal models, human tissues and in vitro studies used to investigate EAT-mediated AF which can contribute to our understanding of cellular and molecular interactions and potentially elucidate new therapeutic targets.

## Introduction

Atrial fibrillation (AF) is the most common clinically relevant arrhythmia, with an estimated global prevalence of approximately 60 million cases^1^. AF is characterised by rapid and irregular activity of the atrial myocardium, resulting in symptoms including palpitations, dyspnoea, dizziness and fatigue. Critically, it is associated with a 5-fold and 2-fold increased risk of stroke and heart failure respectively, significantly increasing morbidity and mortality in the affected population^2–4^. As a result, AF poses a great healthcare and economic burden which is only set to worsen as the prevalence increases with our aging population^5^.

AF is associated with a broad range of risk factors, including diabetes, hypertension, alcohol consumption, age and obesity^6–8^. Obesity is an increasing global epidemic and has been identified as an independent risk factor of AF across many population studies^7,9–11^. It is further suggested that obesity-induced AF is mediated through epicardial adipose tissue (EAT), which contributes to a myopathic atrium susceptible to arrhythmia^7,12,13^. Numerous clinical studies utilising computed tomography and cardiac magnetic resonance imaging show increased EAT thickness in patients with AF^14–16^. Additionally, it has been demonstrated that epicardial fat is a stronger indicator of AF risk compared to overall adiposity, implying that the complexity of EAT is not captured in traditional obesity markers, such as body mass index (BMI)^12,17^. Despite these links between EAT and AF revealed by clinical studies, the precise pathophysiological mechanisms at the molecular and cellular levels remain unclear.

Investigation of the molecular mechanisms that underpin the interplay between obesity, EAT, and AF is well underway. This review will cover how researchers utilise in vitro and in vivo models including human tissue samples, animal models and cell-based models. Developing biologically relevant human models that facilitate high throughput screening assays will prove essential for identifying future drug targets to combat obesity-mediated AF.

## EAT-Mediated Mechanisms of AF

AF is a progressive disease, beginning as paroxysmal AF, characterised by self-terminating intermittent episodes, which can develop into persistent AF, requiring cardiac intervention to restore normal rhythm^18^. Further progression leads to long-standing persistent and permanent AF, where attempts at rhythm control are ceased^18^. The progression of AF is driven by various mechanisms, including the electrical, structural and autonomic remodelling that make up the vulnerable and myopathic atrial substrate. With increased incidence of AF, there is greater remodelling of the atria and increased myopathy which heightens susceptibility to further AF episodes, a phenomenon referred to as “AF begets AF”^19^.

While the mechanisms underlying AF are complex, the fundamental pathophysiology relies on the interplay between a trigger that initiates abnormal electrical activity and a substrate that sustains this activity. Typically, abnormal activity is triggered by ectopic beats, which often originate from within the pulmonary veins, and initiate reentry^20^. However, sustained re-entrant activity requires an appropriate substrate, typically made susceptible through a shortened effective refractory period or slowed heterogeneous conduction, hence increasing patient susceptibility to AF^20^.

## Epicardial Adipose Tissue

**Fig. 1:**
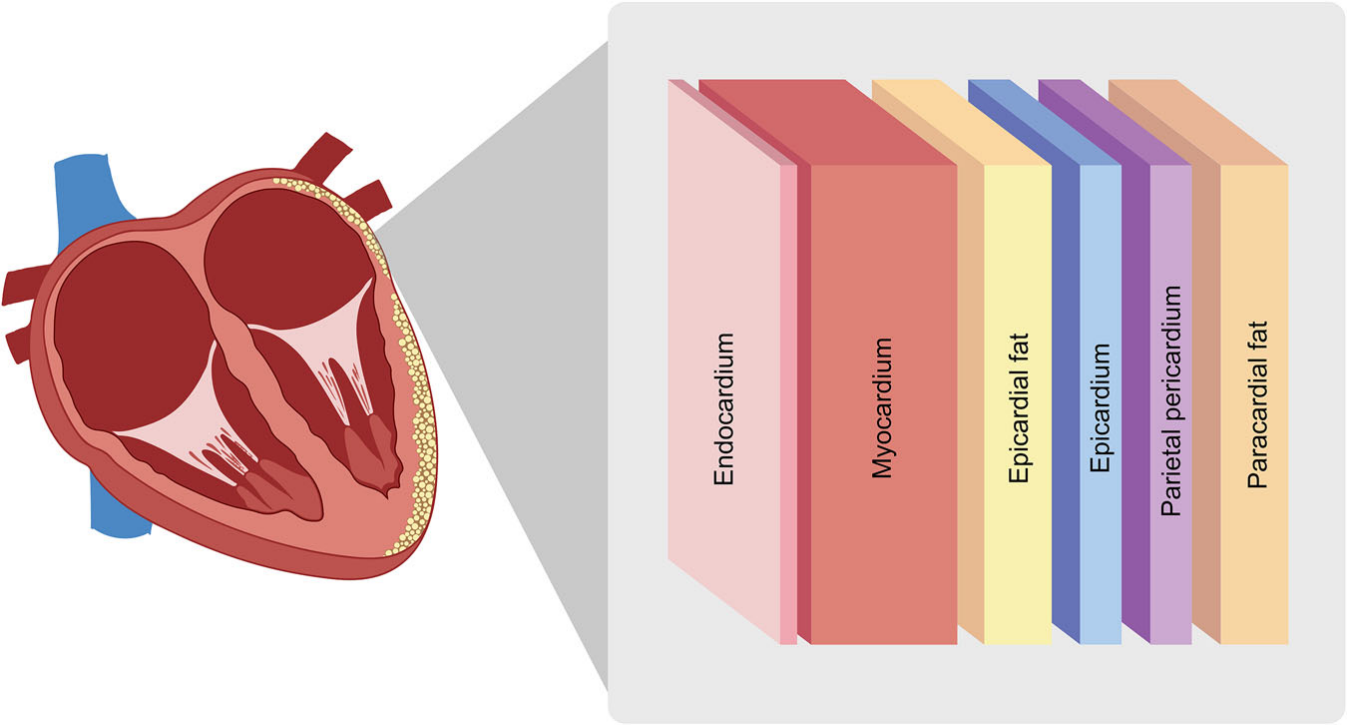
Overview of the anatomical positioning of cardiac fat deposits in relation to the myocardium. Epicardial fat deposits lie in direct contact with the myocardium with no fascia separating the two tissues.

Obesity is associated with an increased volume of EAT, a layer of fat situated between the myocardium and pericardium of the heart (Fig. 1)^21^. This direct contact with the myocardium is unique to EAT, allowing for crosstalk between the two tissue types through shared microcirculation. During development, it is classified as a thermogenic brown adipose tissue that later transitions into a white adipose tissue for energy storage. However, it still maintains beige fat-like properties, demonstrated by increased expression of uncoupling protein 1 (UCP-1) compared to subcutaneous fat^22^. Epicardial fat is made up primarily of adipocytes but also consists of macrophages and other immune cells, nerve cells and stromal cell types^23,24^. Lineage tracing has demonstrated that epicardial adipocytes originate from epicardial progenitor cells that undergo epithelial-mesenchymal transition^25,26^. These precursor cells have the potential to differentiate into either cardiac fibroblasts or adipocytes, committing to an adipogenic fate upon activation of the peroxisome proliferator activated receptor gamma (PPARγ) pathway^26^. Additionally, there is evidence that specific populations of adipose progenitor cells appear with age, potentially contributing to increased accumulation and pathogenesis of EAT^27^.

**Fig. 2:**
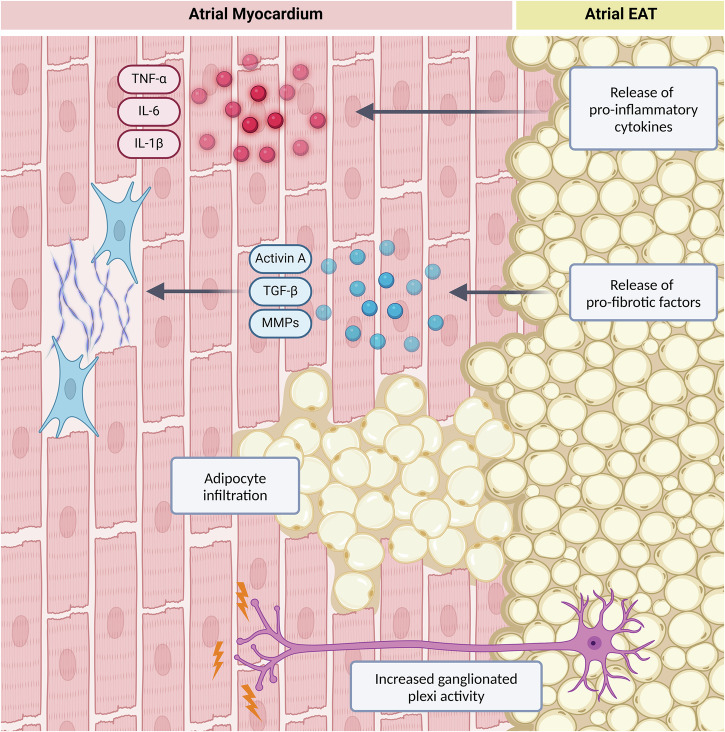
Proposed arrhythmic mechanisms of atrial epicardial fat. Under pathological conditions, epicardial adipose tissue (EAT) can release pro-inflammatory cytokines (tumour necrosis factor (TNF)-α, interleukin (IL)-6 and IL-1β) to induce inflammation in the myocardium, leading to electrical remodelling of the atria. The release of pro-fibrotic factors (transforming growth factor (TGF)-β, activin A and matrix metalloproteinases (MMPs)) can increase extra-cellular matrix production, leading to fibrosis. Infiltration of adipocytes into the myocardium can disrupt cardiomyocyte continuity and cause heterogenous conduction. Autonomic remodelling of the atria can occur via increased activity of the ganglionated plexi located within EAT deposits.

Under normal physiological conditions, EAT plays a cardioprotective role. It secretes anti-inflammatory adipokines, such as adiponectin and omentin, which help reduce inflammation and oxidative stress within the heart and promote insulin sensitivity^28–30^. Additionally, its brown fat-like thermogenic properties provide free fatty acids for energy production whilst also acting as a buffer to protect against excessive fatty acid levels^23,31^. With age, the thermogenic functions of EAT diminish considerably, reducing its protective effects and becoming more pathologically susceptible^32^. Under pathological conditions, epicardial fat can contribute to electrical, structural, and autonomic remodelling of the atria, creating a substrate prone to arrhythmias, as summarised in Fig. 2^23^.

## Electrical Remodelling

Under pathological conditions, EAT undergoes structural and functional changes that result in the overexpression of genes involved in inflammatory processes, leading to increased secretion of proinflammatory cytokines, such as tumour necrosis factor (TNF)-α, interleukin (IL)-6 and IL-1β^30^. In the myocardium these cytokines promote electrical remodelling by modulating ion channel function and calcium handling; specifically, these proinflammatory molecules can stimulate reactive oxygen species production to impair key proteins involved in intracellular Ca^2+^ handling. This leads to spontaneous Ca^2+^ release from the sarcoplasmic reticulum, which in turn activates sodium-calcium exchangers. The resulting depolarising currents can cause delayed afterdepolarisations, ultimately promoting ectopic beats and arrhythmogenesis^33,34^. Additionally, extracellular vesicles (EV) can also be a source of pro-inflammatory factors, demonstrated by the higher levels of TNF-α, IL-1 and IL-6 found in EV samples from AF patients compared to those without AF^35^. These vesicles are secreted from EAT and can transport encapsulated inflammatory molecules to the atrial myocardium, with cardiomyocytes exposed to these EVs in vitro demonstrating shortened APDs and increased susceptibility to re-entrant arrhythmias^35^.

EAT has also been shown to modulate connexin gap-junction expression and localisation which is known to alter atrial electrophysiology and promote AF^36,37^. In excised atrial appendages, patients with increased volumes of epicardial fat show greater lateralisation of connexin-40^37^. This directly affects myocyte electrical coupling, contributing to a proarrhythmic substrate by promoting heterogenous conduction.

## Structural Remodelling

Epicardial fat is also thought to contribute to a myopathic atrium via structural remodelling, specifically through an increase in fibrosis of the atrial myocardium. Patient epicardial fat, but not subcutaneous, has been shown to induce atrial fibrosis in rat atrial sections, with increased secretion of known pro-fibrotic factors including transforming growth factor (TGF)-β, activin A and matrix metalloproteinases (MMPs)^30,38^. TGF-β and activin A promote the activation of fibroblasts to myofibroblasts, resulting in increased ECM deposits and fibrosis^39^. ECM homeostasis is normally regulated by MMPs, however, increased secretion can disrupt this process and lead to the accumulation of fibrotic tissue, leading to heterogeneous atrial conduction and arrhythmogenesis^40^. Furthermore, the epicardium is rich in progenitor cell types capable of differentiating into adipocytes as well as myofibroblasts during pathogenesis^41^. This cellular remodelling can result in further fibrosis and fibro-fatty infiltration into the atrial myocardium, contributing to structural remodelling as seen in AF patients using histological analysis^42,43^.

Another mechanism through which EAT accumulation contributes to structural remodelling involves fatty infiltration into the atrial myocardium. Obese sheep models have demonstrated increased adipose infiltration of myocardial tissue, along with slowed and heterogenous conduction compared to controls^44^. This occurs when excess adipocytes, that arise from resident progenitor cells in the epicardium, intrude into the adjacent atrial myocardium, hence disrupting the alignment of cardiomyocytes. Additionally, free fatty acids released from epicardial fat can infiltrate the myocardium and, in excess, can also decrease cardiomyocyte continuity, leading to conduction abnormalities and physical propagation blockade to promote areas of re-entrant circuits^23^.

## Autonomic Remodelling

Ganglionated plexi, composed of various ganglia and interconnecting axons that innervate sympathetic and parasympathetic nerve fibres, are a normal and abundant component of the intrinsic cardiac nervous system and are typically located within epicardial fat deposits^45,46^. Dysfunctional or excessive EAT accumulation can lead to increased activity of these ganglia, resulting in both sympathetic and parasympathetic stimulation. Sympathetic nerve activation has been shown to increase calcium transients within myocytes, while parasympathetic activation causes a reduction in the atrial refractory period and promotes EAD triggers, both contributing to an AF substrate^47,48^. It is proposed that this is mediated through increased EAT release of inflammatory mediators or an excess of free fatty acids that may activate the autonomic nervous system through an increase in local catecholamine levels^49,50^. While direct molecular links between EAT and autonomic dysfunction are unclear, evidence supports an EAT-mediated effect. Suppression of nearby ganglionated plexi through methods like ablation or botulinum toxin injection into epicardial fat pads is associated with reduced arrhythmogenesis and AF recurrence, suggesting EAT mediates autonomic dysfunction^51^.

## Methods to investigate effects of EAT

The use of models is crucial for investigating underlying disease mechanisms and informing the development of new treatment options. Clinical and epidemiological studies of obese patient cohorts have provided extensive knowledge on the correlation between EAT and AF incidence^7,12,52^. However, practical and ethical restraints make it difficult to investigate current knowledge gaps, including the mechanistic effects that occur on a molecular, cellular and tissue level. As a result, researchers use alternate methods to model EAT and AF, including animal models, samples from human tissue and other in vitro models, as summarised in Table 1. To identify effective drug targets within these mechanisms, it is essential to develop advanced models that mimic human EAT and its effects on the heart.

**Table 1:** Overview of the strengths, weaknesses and insights gained from different models of EAT-mediated AF

Model Type		Strengths		Limitations	Insights Gained on EAT-AF Pathophysiology
**Animal Models**	**Rodents**	Low-cost animal model; easily genetically modifiable.	Lack of substantial epicardial fat that replicates human EAT anatomical location; differing atrial electrophysiology compared to humans^64^.		Mouse pericardial fat alters cardiomyocyte conduction and has a distinct proteome compared to subcutaneous fat (e.g. increased abundance of focal adhesion proteins)^37^. Increased volume of EAT in guinea pigs provides a useful model for studying paracrine-related effects^55^. Limited EAT-specific studies relating to AF.
	**Rabbits**	Increased similarity to human EAT deposition and atrial electrophysiology compared to rodents^64^.	Lower throughput model compared to rodents.		Demonstrated increased atrial arrhythmogenesis and altered cell morphology in a model of heart failure^53^.
	**Pigs**	High similarity to human EAT anatomy and atrial electrophysiology.	High cost and challenging to maintain; low throughput.		AF induction in vivo results in differential expression of genes relating to oxidative stress and tissue remodelling^103^. Widely used in studies on ablation techniques and the effect of EAT due to similarities in anatomy to human heart^104–106^.
	**Sheep**	Similar to human EAT anatomy and atrial electrophysiology; able to simulate chronic AF^64,107^.	High cost and challenging to maintain; low throughput.		Obese sheep show increased EAT infiltration into atrial myocardium with altered conduction, fibrosis and arrhythmic activity^44^.
**Human Tissue Models**	**Human Adipose Tissue**	Highly relevant human model that encompasses whole physiological environment.	Limited availability of fresh tissue; often of unhealthy patient origin meaning limited healthy control samples.		Tissue profiling reveals unique transcriptome and secretome of EAT compared to subcutaneous fat, including an increase in proinflammatory cytokines (TNFα, IL6 and IL-1β) and pro-fibrotic factors (TGF-β, activin A, MMPs).^30,38,71^
	**Isolated Adipocytes**	Maintains relevant human EAT characteristics.	Difficult to source and limited scalability; lacks complete environment of EAT.		Limited studies focus on isolated adipocytes from EAT to investigate AF.
**In Vitro Models**	**3T3-L1 Cell Line**	Well established cell line with simple protocol to produce adipocytes in high efficiency.	Lacks human and epicardial-like specificity.		Instrumental in understanding adipogenesis in general; used to validate inflammatory markers seen in EAT associated with AF, such as IL-6, TNF-α and TGF-β1^103,108^.
	**Primary MSCs and ADSCs**	Maintains human adipocyte characteristics; differentiate to adipocytes with high efficiency.	Primary MSCs have limited scalability; require invasive donor procedures; differentiated adipocytes lack EAT-like specificity.		Differentiated adipocytes from ADSCs secrete adipokines associated with AF (e.g. MCP-1 and IL-6) and alter electrophysiology of iPSC-cardiomyocytes^78^.
	**iPSCs**	Potentially unlimited source of adipocytes; potential for isogenic cocultures with other differentiated cell types; facilitates patient-specific studies; relevant human model.	Current protocols to differentiate adipocytes lack efficiency, maturity and specificity to EAT-like adipocytes^81,83^.		Limited studies use iPSC-derived adipocytes to study AF, some research related to cardiometabolic disease^109^.

## Animal Models

**Fig. 3:**
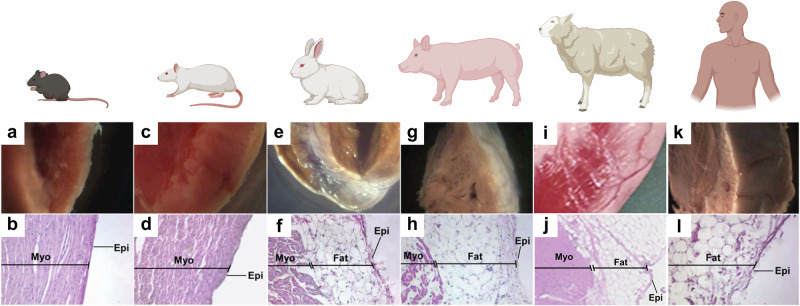
Distribution of ventricular epicardial fat across different species. Mice (**a**, **b**) and rats (**c**, **d**) have limited amounts of EAT. Rabbits (**e**, **f**), pigs (**g**, **h**) and sheep (**i**, **j**) have a more similar distribution of EAT compared to humans (**k**, **l**). Images (**a**–**h**, **k**, **l**) reproduced from Yamaguchi et al. *PNAS (2015)*, under a Creative Commons Attribution license^26^. Images (**i**, **j**) reproduced with permission from Mahajan R et al. *JACC (2015)*, © 2015 American College of Cardiology Foundation^44^.

Researchers have employed various animal models to study the role of epicardial fat, including rodents, rabbits, pigs and sheep (Fig. 3)^26,44,53^. Mice and rats have limited amounts of EAT, which is primarily confined to the atrio-ventricular groove of the heart^26^. This limits their capacity in replicating how human epicardial fat, which lies in direct contact with the myocardium of the atria and ventricles, influences electrical and structural cardiac function^23^. However, it has been shown that murine pericardial fat can modulate cardiomyocyte conduction properties and has a distinct proteome with an increased expression of focal adhesion proteins^37^. Guinea-pigs may provide an improved rodent model due to their increase in EAT volume that acts as a rich source of cytokines similarly found in human EAT, including TNFα, adiponectin and IL-1β^54,55^. However, their epicardial fat primarily accumulates around the aortic arch and coronary arteries, with limited direct contact with the atrium, though they remain useful for explant studies examining paracrine effects^54,55^.

Larger animals, however, display epicardial fat distribution in a comparable manner to the human heart, making them a more accurate animal model for studying obesity-related cardiac conditions^26^. Sheep and pig models have been heavily utilised in cardiac research due to their similarities in size and physiology compared to the human heart. They can also be used to examine the role of EAT in heart disease by mimicking human obesity conditions through high-caloric diets that stimulate increased epicardial fat accumulation. Mahajan et al. demonstrated that obese sheep had an increase in epicardial fat infiltration into the atrial myocardium, resulting in conduction abnormalities, increased fibrosis and likelihood of atrial arrhythmias^44^. Likewise, another obese sheep model demonstrated increased lipidosis and fibrosis of the left atrium, accompanied by increased conduction heterogeneity and susceptibility of arrhythmias^56^. Rapid atrial pacing can be used to induce AF in sheep, with these models not only showing increased fatty infiltration into the atria alone but also increased fibrosis of this fatty tissue compared to control sheep, suggesting fibro-fatty infiltration to be a key determinant of EAT-mediated AF^43^. These sheep models can recapitulate what is implicated in human clinical studies and uncover novel pathological processes related to EAT-mediated AF mechanisms. Pigs also have comparable levels of epicardial fat to humans and have been used in studies assessing the effects of diet and exercise on the inflammatory profiles and fatty acid composition of EAT^57–59^. While these studies do not focus specifically on AF, they still provide insights into how lifestyle factors may influence epicardial fat characteristics, with broad implications for cardiovascular diseases in general.

Ex vivo experiments can also be performed using animal tissue. Acute atrial slices with EAT attached can be excised and collected, maintaining the native environment and secretions. While optical mapping of rat hearts ex vivo has revealed increased AF susceptibility in both aged and high-fat diet fed rats, there has been no focus on epicardial fat specifically^60,61^. Additionally, cardiac fat-conditioned media from animal models can be applied to separate cardiac tissue slices or cardiomyocytes to assess the paracrine effects of secreted factors^55,62,63^. For example, it was shown that secreted factors from guinea pig EAT altered calcium handling in rat cardiomyocytes^55^. Further investigation of how these secreted factors influence conduction and electrophysiology can provide insights into potential mechanisms of EAT-induced atrial remodelling.

Using larger animal models to research the role of epicardial fat on AF may be valuable in pre-clinical screening tests due to their relevance to the human heart. However, this approach does not provide sufficient throughput for target and drug screening assays in a cost-effective manner. Additionally, AF does not typically occur naturally in animals, meaning it must be induced by external triggers^64^. This complicates the study of AF initiation and progression, as it introduces experimental variables that may not completely replicate the spontaneous development of AF as seen in humans^64^. Overall, while animal models can be used to provide valuable insight into the effect of EAT on AF, higher throughput and more cost-effective models are needed to probe the mechanisms driving myopathic remodelling to identify potential therapeutic targets.

## Human Tissue Models

### Epicardial fat tissue

To investigate the role of epicardial fat in AF, the most accurate models will inevitably involve the use of human tissue, such as human atrial tissue slices or isolated atrial cardiomyocytes^65,66^. While these models provide an accurate human atrial electrical and structural phenotype, atrial tissue is challenging to obtain, and the non-proliferative nature of cardiomyocytes makes them a limited resource. Similarly, human EAT samples are difficult to source, with live tissue only able to be obtained from patients undergoing cardiac surgery. As a result, most available samples are from myopathic sources or tainted with fat-soluble anaesthetic, offering limited access to non-diseased control tissue for comparison.

Despite this, numerous studies have utilised epicardial fat biopsies to conduct in-depth profiling of EAT, revealing its unique transcriptome and secretome compared to subcutaneous and other visceral fat deposits^30,38,67,68^. Findings from these studies have provided valuable insight into the potential mechanisms in which EAT can mediate AF, as discussed above. However, very few studies have explored the direct effect of human EAT on cardiac cell types to identify a direct link to cardiovascular disease, or AF specifically.

### Human EAT secretions

The paracrine effects of EAT can be investigated using human EAT-conditioned media added to cardiomyocytes. Currently, such studies primarily focus on endothelial cells and vascular effects, rather than atrial myocardium function^69,70^. EVs can also be studied to investigate the effect of EAT secretions. Shaihov-Teper et al., reported that isolated EVs from the epicardial fat of AF patients had increased expression of proinflammatory and profibrotic cytokines, compared to those without AF^35^. These cytokines included IL-1α, IL-6, TNF-α, all associated with the pathogenesis of AF^23,37,71^. Incubation of the AF patient EVs with cardiomyocytes induced inflammation, fibrosis and increased sustained rotor formation, suggesting a direct link between EAT and atrial myopathy^35^.

### Isolation of human EAT adipocytes

Alternatively, epicardial adipocyte cells can be directly incorporated into co-culture systems with isolated or differentiated cardiomyocytes. Quarta et al. demonstrated an optimised protocol for isolating “ready-to-use” mature adipocytes, potentially adaptable to epicardial-specific fat deposits^70^. The limited studies using these human tissue approaches stem from challenges like obtaining live samples, the non-proliferative nature of adipocytes making it a limited resource, and their low throughput capacity. While highly relevant for understanding the role of EAT in AF in the human heart, these approaches require complementary scalable models that facilitate large-scale target and drug discovery to assist in the development of new AF therapies.

## In Vitro Models

**Fig. 4:**
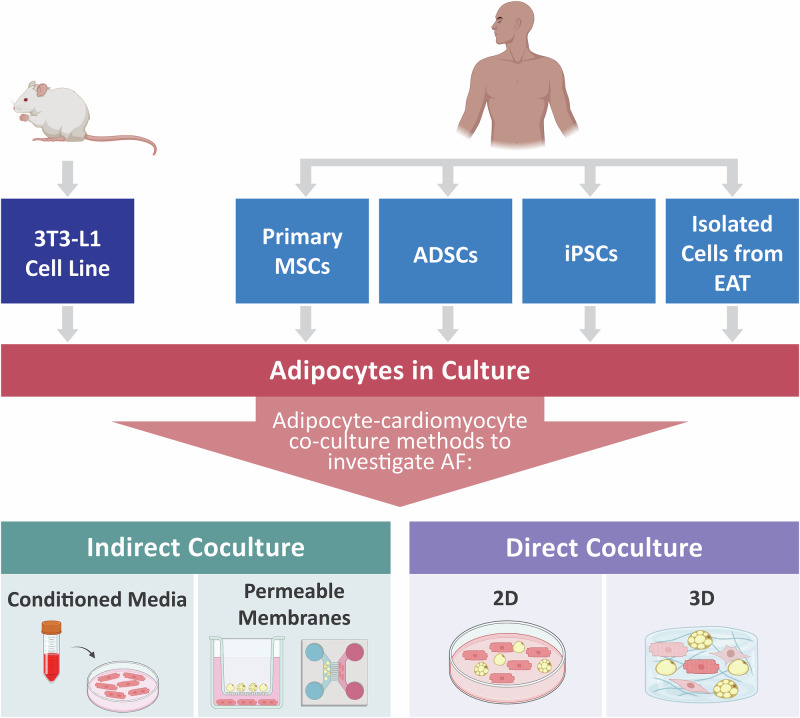
Overview of different in vitro approaches to study EAT-mediated AF. Cultured adipocytes can be derived from numerous sources including the murine 3T3-L1 cell line, human primary MSCs, ADSCs, iPSCs or isolated cells from epicardial fat biopsies. To study AF, these adipocytes can be cocultured with cardiomyocytes and their cardiac cell types using both direct and indirect methods.

Another approach for modelling the influence of EAT on AF is the use of in vitro studies, which can utilise a range of cell sources and co-culture techniques to probe arrhythmic mechanisms at a cellular and molecular level, as outlined in Fig. 4.

### Cell sources

Epicardial fat is primarily made up of adipocytes and can be differentiated from a range of cell sources. The murine 3T3-L1 cell line has been traditionally used to model adipogenesis due to their propensity to differentiate into adipocytes^72,73^. Established cell lines offer a reproducible and cost-effective approach to model adipose tissue but display physiological differences to human adipocytes, lacking the complexity and specificity of EAT. Human cell sources, including adipose-derived stem cells (ADSCs) and primary mesenchymal stem cells (MSCs), can be differentiated to adipocytes as well^74,75^. While bone marrow-derived MSCs have limited proliferation capacity, human ADSCs can be cultured for multiple passages and maintain adipocyte differentiation potential post-cryopreservation^76^. Additionally, ADSCs can be differentiated into adipocytes with relatively high efficiency and lipid droplet size when compared to other human MSC sources^77^. However, these cells represent a more WAT or subcutaneous fat-like profile and lack specificity to epicardial fat-like adipocytes, as demonstrated by the lack of UCP-1, a brown-fat expression marker^78^.

Human induced pluripotent stem cells (hiPSCs) offer an alternative approach to study epicardial fat and its role in AF. HiPSCs have the ability to differentiate into all somatic cell types without possessing the ethical concerns associated with embryonic stem cells. They therefore provide a potentially limitless source of adipocytes and cardiomyocytes to study EAT-mediated AF in an isogenic system. IPSCs also retain the donor’s genetic makeup enabling focused patient-specific or wide-level population-based studies to explore both individual and group variations associated with AF^79,80^.

Over the past decade, researchers have developed protocols for differentiating iPSCs into adipocytes. The standard method involves first differentiating cells to MSCs, followed by induction of adipogenesis through the addition of a cocktail of factors shown to activate known adipogenic signalling pathways^81^. However, this method can result in poor maturity, characterised by small lipid droplets, and poor differentiation efficiency, requiring the need for further purification steps^81–83^.

Alternatively, iPSC-MSCs can be differentiated to adipocytes by virally inducing expression of PPARG2, a known master regulator of adipogenesis^84^. This protocol, developed by Ahfedlt et al. was able to produce white adipocytes with high specificity and an efficiency of 88%^84^. However, genetic manipulation via viral transduction cannot recapitulate the natural process of adipogenesis that occurs in human development and increases the risk of mutagenesis. Additionally, these methods do not produce epicardial-like adipocytes, which are known to have a unique transcriptional profile distinct from subcutaneous fat and other visceral fat types^30,67^. Specifically, EAT has a beige fat-like profile, expressing brown fat (UCP-1, Cidea, PPAR gamma co-activator 1 alpha) and some white fat (leptin, homeobox C9) markers, as well as the beige-fat specific marker CD137^22,30^. Further development of differentiation protocols is needed to address this drawback.

### Co-culture techniques

To model the interplay between EAT and AF, differentiated adipocytes can be utilised in various co-culture systems with other differentiated cardiac cell types, mainly cardiomyocytes. The differentiation of iPSCs into cardiomyocytes is well established, with protocols capable of generating pure and robust populations with atrial-like phenotypes^85–87^. These cardiomyocytes enable researchers to investigate cellular mechanisms associated with AF by inducing and measuring changes in action potentials, Ca^2+^ handling, conduction velocities and propagation irregularities^85,86^.

### Indirect co-culture systems

The paracrine effects of adipocyte secretions can be investigated by subjecting cardiomyocytes to conditioned media from adipocytes. Secretions from both ADSC-derived adipocytes and murine adipocytes have been shown to contain significant levels of pro-inflammatory and pro-fibrotic cytokines^37,78^. Independent addition of these adipokines to cardiomyocytes result in changes in conduction and electrophysiology^78^.

Additionally, microfluidic systems can facilitate controlled paracrine signalling between the two cell types to recapitulate the crosstalk that occurs between the EAT and myocardium. This system allows for physical separation of cell types to accommodate specific medium requirements, while assessing the impact of adipocyte-derived secretions on cardiomyocyte properties. This method has been used to study macrophage-induced inflammatory effects on cardiomyocytes and presents a potential tool for studying adipocyte-related effects despite limited use in the current literature^88^.

### Direct co-culture systems

Another approach is an integrated co-culture model, with adipocytes dispersed among cardiomyocytes to simulate adipocyte infiltration of the atrial myocardium. These models can be developed in either 2D or 3D formats. A study by Morrissette-McAlmon et al. using this model in 2D showed slowed conduction velocity, altered action potential properties and altered calcium handling in their cocultures, however, this was conducted with ventricular cardiomyocytes^78^. While 2D monolayer systems allow for a simplified yet effective model, they fail to recapitulate the native architecture and complex environment of human atrial tissue, hence, significant research has been done to advance and develop 3D models. While 3D organoid and engineered tissue models using adipocytes have been developed to investigate effects of macrophages and vascularisation, there is no current literature, to our knowledge, on the use of adipocytes and cardiomyocytes in a 3D co-culture system^89,90^.

### Future directions

To study various heart diseases, hiPSC-derived cardiomyocytes can be cultured in 3D, either as self-assembling organoids or by seeding the cells into designed scaffolds to produce engineered heart tissues (EHTs)^91^. These structures have been shown to have enhanced structural, electrical and metabolic maturity compared to their two-dimensional counterparts. Specifically, they show enhanced parallel alignment of myofibrils, improved Ca^2+^ signalling, more physiological APDs and changes in energy metabolism^92–94^. 3D structures facilitate complex interactions between numerous cell types and extracellular matrix components. For example, the incorporation of cardiac fibroblasts promotes intercellular interactions via gap junction formations and additionally enhances maturation^95^. Endothelial cells have been shown to promote vascularisation within 3D tissue, improving the functionality and maturity of the cardiomyocytes^96,97^.

In EHTs, various differentiated cell types can be combined in specific ratios to represent physiological and pathological cell stoichiometries associated with different conditions, making them valuable for disease modelling and drug testing. For example, the incorporation of increasing percentages of adipocytes can represent an increasingly obese model with more fat infiltration. Additionally, incorporating immune cell types that reside within EAT, such as macrophages, can allow for a more complete recapitulation of the inflammatory processes that occur at the interface of the atrial myocardium, albeit at the risk of exceeding the practicality and scalability needed for a high throughput model^89^. At present, there is minimal use of 3D cultures of atrial-specific cardiomyocytes, especially with the inclusion of adipocytes. Instead, most 3D cardiac tissue models rely on mixed or ventricular populations, which are often used to model ventricular arrhythmias, such as Torsades de Pointes^98^. Additionally, many of the current scaffolds and formations used in these models would not allow for the observation of re-entrant arrhythmias due to their limited spatial area. This highlights the need for optimisation in this area to effectively study AF in a 3D culture system.

Another in vitro limitation that requires addressing is the generation of EAT-like specific adipocytes. As previously mentioned, epicardial fat has a distinct profile compared to subcutaneous fat, including more beige fat-like characteristics and pro-inflammatory secretory profile^22,30,38^. A potential method to generate EAT-like adipocytes is to exploit the adipogenic potential of fibroblasts to differentiate into adipocytes^99^. Additionally, information gained from lineage tracing of human epicardial adipocytes can guide the development of methods to differentiate them more specifically in vitro. During human embryonic development, EAT is produced from epicardial and endocardial progenitor cells that undergo epithelial to mesenchymal transition to produce epicardial fat tissue^25,26^. Utilising an existing protocol that generates cardiac-like fibroblasts via an epicardial or endocardial pathway and then proceeding to add an adipogenic cocktail may promote the differentiation of EAT-like adipocytes^100,101^. Furthermore, utilising secreted factors from atrial myocytes that have known adipogenic potential, such as atrial natriuretic peptide, may produce adipocytes with greater epicardial characteristics^25^. This method aims to mimic the natural development of EAT and provide an iPSC-derived cell model to study obesity-related AF that recapitulates human cardiac physiology. Overall, the differentiation of adipocytes from iPSCs and subsequent coculture methods with cardiomyocytes can provide insight into EAT-mediated structural and electrical remodelling in atria. Additionally, iPSCs have the potential for scalability and high throughput experimentation to facilitate drug discovery. However, the inherent immature phenotype of iPSC-derived cell types presents a limitation; for example, iPSC-derived cardiomyocytes exhibit foetal-like structural features, electrophysiology and metabolism^102^. This immaturity, compared to human adult cardiomyocytes, can hinder their applications in accurately modelling AF, meaning human tissue and larger animal models remain essential pieces for replicating the complete native cellular environment and structural architecture of the atrial myocardium and adjacent EAT. If findings from these more accurate but expensive models can be validated in in vitro systems, they can act as complementary approaches to inform target and drug screening assays for novel AF therapeutics.

## Conclusion

The rising prevalence of both obesity and atrial fibrillation highlights an urgent need for models that replicate the human cardiac environment, particularly in the context of EAT-mediated AF. Here, we have provided an overview of the different approaches used to study mechanistic links between EAT and AF, including animal studies, human tissue and in vitro models. High throughput models that balance complexity with scalability are needed to facilitate the discovery of upstream pathways that have the potential to serve as therapeutic targets. Despite current limitations, continued development and application of these diverse model types are crucial for enhancing our understanding of EAT-mediated AF and informing future treatment options.

## Data Availability

No datasets were generated or analysed during the current study.
